# Reducing Medication Errors by Adopting Automatic Dispensing Cabinets in Critical Care Units

**DOI:** 10.1007/s10916-023-01953-0

**Published:** 2023-04-27

**Authors:** Hui-Ning Tu, Tzu-Hao Shan, Yu-Chin Wu, Pei-Hsuan Shen, Tsung-Yu Wu, Wen-Liang Lin, Yea-Huei Yang-Kao, Ching-Lan Cheng

**Affiliations:** 1 Department of Pharmacy, National Cheng Kung University Hospital, College of Medicine, National Cheng Kung University, Tainan, Taiwan; 2 School of Pharmacy, Institute of Clinical Pharmacy and Pharmaceutical Sciences, College of Medicine, National Cheng Kung University, Tainan, Taiwan; 3 Health Outcome Research Center, National Cheng Kung University, Tainan, Taiwan

**Keywords:** Automated dispensing cabinet, Medication error, Intensive care unit, Taiwan, Pharmacist

## Abstract

Medication errors can have severe consequences and threaten patient safety. The patient safety-related benefits of automated dispensing cabinets (ADCs) have been reported by several previous studies, including a reduction in medication errors in intensive care units (ICUs) and emergency departments. However, the benefits of ADCs need to be assessed, given the different healthcare practice models. This study aimed to compare the rates of medication errors, including prescription, dispensing, and administrative, before and after using ADCs in intensive care units. The prescription, dispensing, and administrative error data before and after the adoption of ADCs were retrospectively collected from the medication error report system. The severity of medication errors was classified according to the National Coordinating Council for Medication Error Reporting and Prevention guidelines. The study outcome was the rate of medication errors. After the adoption of ADCs in the intensive care units, the rates of prescription and dispensing errors reduced from 3.03 to 1.75 per 100,000 prescriptions and 3.87 to 0 per 100,000 dispensations, respectively. The administrative error rate decreased from 0.046 to 0.026%. The ADCs decreased National Coordinating Council for Medication Error Reporting and Prevention category B and D errors by 75% and category C errors by 43%. To improve medication safety, multidisciplinary collaboration and strategies, such as the use of automated dispensing cabinets, education, and training programs from a systems perspective, are warranted.

**Supplementary Information:**

The online version contains supplementary material available at 10.1007/s10916-023-01953-0.

## Background

Medication errors are defined as preventable events that lead to inappropriate medication use or patient harm, which may occur during prescription, dispensing, or administration [1]. Medication errors can have severe consequences and threaten patient safety. Seven thousand to 9000 people die due to medication errors, and more than 7 million people in the USA are affected and need more than $40 billion to receive each year [2]. In recent decades, several hospitals have adopted computerized provider order entry systems for prescribing assistance and barcode medication administration systems for patient and medication identification to reduce medication errors [3]. The core role of pharmacists related to medication safety is to ensure optimal prescriptions and appropriate dispensing of medications to patients. Common strategies for preventing dispensing errors include dispensing alerts such as tall-man letters, dosage form notifications on each pack for each confusable medicaiton, and double-checks before delivery. However, with alerting measures, the dispensing process mostly relies on human operations and remains an error-prone procedure. Consistent with the concept of the closed-loop electronic medication management system, automated dispensing cabinets (ADCs) are devices that control medication storage, management, dispensing, and delivery that may integrate human operations with electronic information and allow tracking of every medication-related issue throughout the medication process [4].

The patient safety-related benefits of ADCs have been reported by several previous studies, including a reduction in medication errors in intensive care units (ICUs) and emergency departments [5–8]. However, the benefits of ADCs still need to be assessed, given the different healthcare practice models. ADCs will change the workflow of pharmacists and nurses and integrate with original procedures and practices. Some countries, such as the USA and Canada, have authorized pharmacy technicians to dispense and prepare ready-to-administer syringes for intravenous medications. The introduction of ADCs to ICUs and emergency departments in the USA has increased the workload of pharmacy technicians but changed the workflow of nurses [9]. In contrast, only pharmacists are allowed to dispense in Asian countries such as Japan and Taiwan, and nurses prepare medications before administering them to patients [10].

In addition, ADCs should be considered to ensure staff safety, develop complete scheduling, and maintenance of medication inventory during pandemics such as coronavirus disease 2019 [11]. Therefore, this study aimed to assess the benefits of ADCs by comparing the rates of medication errors, including prescription, dispensing, and administrative, before and after their adoption in a medical center in Taiwan.

## Methods

### Setting

The study was conducted at the ICU of the National Cheng Kung University Hospital (NCKUH), a medical center in southern Taiwan with 1200 beds. Since 2005, physician prescriptions have been sent to the pharmacy for dispensing through a computerized physician order entry system and integrated into the electronic medical record system at NCKUH. Medications were delivered daily with medicine trolleys by orderly from the pharmacy to the ICU. In June 2020, ADCs (Pyxis Medstation, BD) were introduced to the ICU of the NCKUH. Seven ADC sets were installed in the ICUs, including the medical, surgical, and pediatric units.

### Design

The medication errors before and after the introduction of ADCs were compared. Two different periods of 6 months each were compared: September 2019 to February 2020 (pre-ADC period) and September 2020 to February 2021 (post-ADC period). The study protocol was reviewed and approved by the Institutional Review Board of NCKUH (B-ER-111-298).

### Workflow for Pre- and Post-interventions

**Fig. 1:**
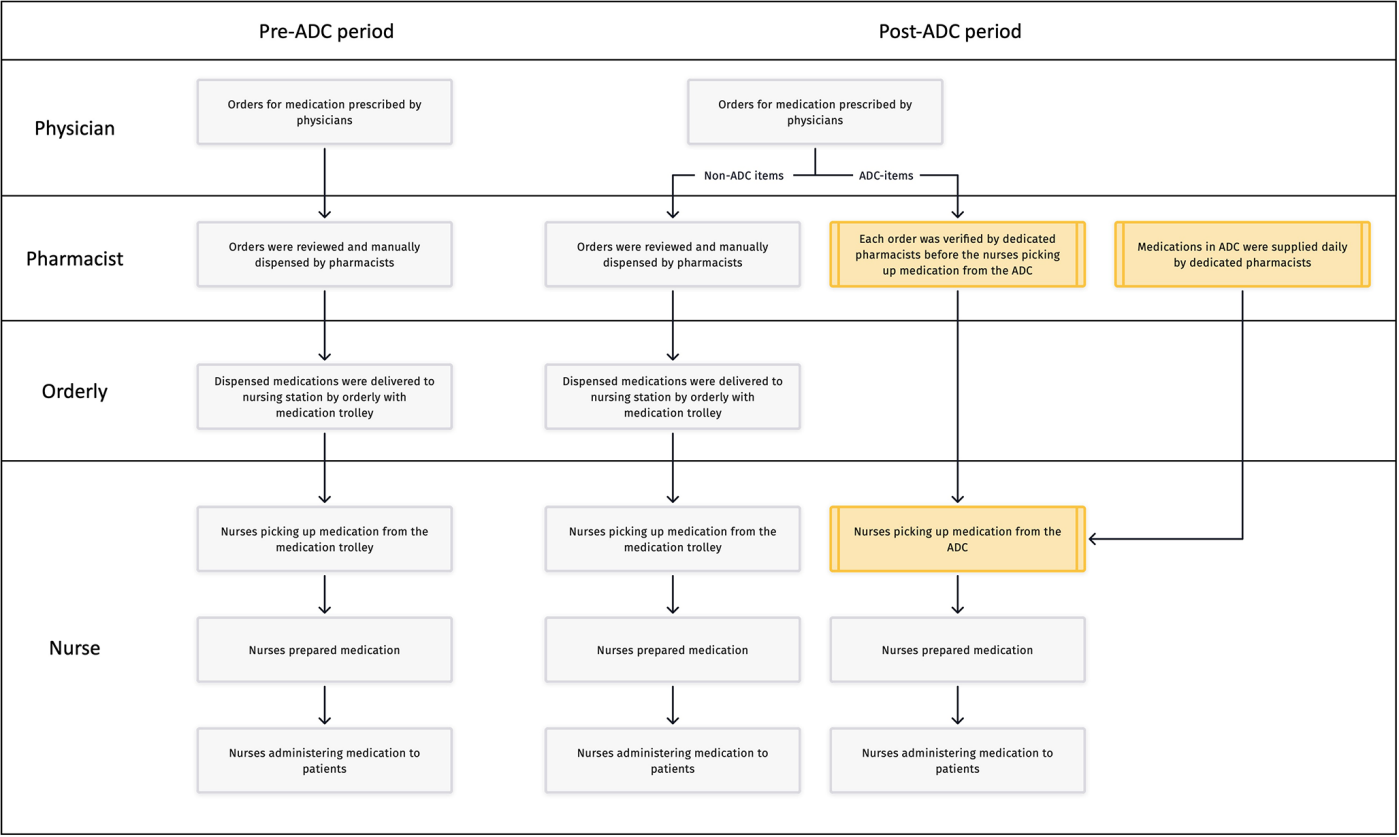
Workflow for the pre-ADC and post-ADC periods. ADC, automatic dispensing cabinet

Each set of ADCs was installed in the nursing station, and directly connected to the electronic medical record of the hospital. A total of 760 medications was prescribed in these ICUs, and 253 were selected as ADC items. Regarding the capacity of ADC, we selected the top 80% of medications based on the consumption and prescription frequency to ensure that the most needed items were stored in the ADC. On the other hand, medications that were classified as the controlled substances or required refrigerator storage were excluded due to the hardware limitation. The adoption of ADCs brought changes to the workflow, except for the prescription process. Pharmacist labor time had a better allocation and allow to verify all prescriptions before nurses picked up medications from the ADCs. In addition, there were other designated pharmacists responsible for supplying ADC medications. For nurses, the main difference was the location for picking up the medications. ADC items could only be picked up 6 h before and after the administration time. The workflow for medications not stored in the ADC remained the same as before for pharmacists and nurses. The workflow at baseline and with the ADCs is shown in Fig. 1.

### Data Collection

The prescription, dispensing, and administrative error data were retrospectively collected from the medication error reporting systems operated by the Committee of Quality Control of NCKUH, Department of Pharmacy, and the Department of Nursing. These errors were mainly reported by the nurses. Prescription errors in the NCKUH report system are defined as errors not identified before delivery. Dispensing and administrative errors were defined as any discrepancies during the processes between prescription and medication use (dispensing, picking, preparation, and administration).

### Study Outcomes

The study outcome was the rate of medication errors, including prescription, dispensing, and administrative. Medication error definitions were previously provided by Barker in a study that identified the incidence of medication errors among 36 healthcare facilities (see 2) [12]. Since not all medications were stored in the ADCs, we calculated the medication error rates for all items and ADC items. We further classified the severity of medication errors for the ADC items. The severity of medication errors was evaluated by two pharmacists according to the National Coordinating Council for Medication Error Reporting and Prevention (NCC MERP) method (see Additional File 3) [13]. If there was any disagreement between the two reviewers, a resolution was reached by consensus after discussing the case with a third pharmacist.

### Statistical Analysis

Categorical data, such as the number of error events, are expressed as frequency and proportion. The rate of medication errors was calculated as follows:

$$\begin{aligned}& Prescribing\,error\,rate\,\left(per\,\text{100,000}\,prescriptions\right)\\ & \quad =\frac{\text{Number}\,\text{of}\,\text{prescription}\,\text{error}\,\text{events}}{\text{Number}\,\text{of}\,\text{prescription}\,\text{orders}} *\text{100,000}\\ & Dispensing\,error\,rate\,\left(per\,\text{100,000}\,dispensing\right)\\ & \quad=\frac{\text{Number}\,\text{of}\,\text{dispensing}\,\text{error}\,\text{events}}{\text{Number}\,\text{of}\,\text{medication}\,\text{dispensing}}*\text{100,000}\\ & Administration\,error\,rate\,\left(\%\right)\\ & \quad=\frac{\text{Number}\,\text{of}\,\text{administrative}\,\text{error}\,\text{events}}{\text{Total}\,\text{inpatient}\,\text{day}}*100\% \end{aligned}$$

During the post-ADC period, pharmacists supplied medications to the ADC instead of dispensing every prescription or medication order. Therefore, the number of medication dispensations for all items was estimated from the ratio of prescriptions to dispensations during the pre-ADC period. Fisher’s exact test was used to compare the medication error rates during the pre- and post-ADC periods. Statistical significance was set at p < 0.05. The data were analyzed using SPSS Statistics version 17.0.

## Results

### Setting Characteristics

The major service utilizations of the ICU are listed in Table 1. The average durations of hospital stay during the pre- and post-ADC periods were 8.2 and 8.7 days, respectively. The number of admissions, prescriptions, and dispensations of medicines was similar during both periods.

**Table 1:** Characteristics of wards with ADCs

	Pre	Post
Number of beds (N)	116	116
Occupancy percentage (%)	93.4	92.4
The average length of stay (day)	8.2	8.7
Number of admissions (N)	2184	2042
Total inpatient day (N)	19,770	19,443
Number of prescription orders^*^ (N)	164,794	171,121
Number of dispensations (manual + ADC) (N)	206,728	215,421^**^

ADC: automatic dispensing cabinet

Pre: pre-ADC period, Post: post-ADC period

^*^One order was counted as one medication

^**^Number of manual and ADC dispensations during the post-ADC period was estimated from the prescription-dispensing ratio during the pre-ADC period

### Medication Error Rate

For all items, five prescription, eight dispensing, and 17 administrative errors were identified during the pre-ADC period, accounting for 3.03 per 100,000 prescriptions; 3.87 per 100,000 dispensations; and 0.09% of administrations, respectively. No dispensing errors were reported during the post-ADC period. Three prescription errors and 22 administrative errors were reported, with error rates of 1.75 per 100,000 prescriptions and 0.11% during the post-ADC period (Table 2).

**Table 2:** Frequencies of medication errors during the pre- and post-ADC periods for all items

Type of medication error	Pre	Post	P-value
Prescription	5	3	0.500
Wrong dose	3	3	
Wrong frequency	1	0	
Wrong form	1	0	
Dispensing	8	0	0.003*
Unauthorized drug (Wrong drug)	4	0	
Wrong dose	2	0	
Wrong form	2	0	
Preparation/Administrative	17	22	0.426
Unauthorized drug (Wrong patient, wrong drug)	4	7	
Wrong dose	9	7	
Wrong route	0	2	
Wrong time	4	6	
Total	30	25	

ADC, automatic dispensing cabinet

Pre: pre-ADC period, Post: post-ADC period

Chi-squared test, *p < 0.05

For ADC items, two prescription, four dispensing, and nine administrative errors were identified during the pre-ADC period, accounting for 1.87 per 100,000 prescriptions, 2.45 per 100,000 dispensations, and 0.046% of administrations. No dispensing errors were reported during the post-ADC period. One prescription and five administrative errors were reported, and the error rates were 0.91 per 100,000 prescriptions and 0.026% during the post-ADC period (Table 3). We further evaluated the errors related to medication prescription and administration that could not be prevented by the ADC (see Additional File 4).

**Table 3:** Frequencies of medication errors during the pre-and post-ADC periods for ADC items

Type of medication error	Pre	Post	p-value
Prescription	2	1	0.620
Wrong dose	2	1	
Dispensing	4	0	0.057
Unauthorized drug (Wrong drug)	2	0	
Wrong dose	2	0	
Preparation/Administrative	9	5	0.424
Unauthorized drug (Wrong patient, wrong drug)	1	2	
Wrong dose	7	0	
Wrong route	0	1	
Wrong time	1	2	
Total	15	6	

ADC, automatic dispensing cabinet

Pre: pre-ADC period, Post: post-ADC period

Chi-squared test, *p < 0.05

### Severity of Medication Errors

All ADC medication errors during the two study periods were classified as errors that caused no harm (categories B to D) (Table 4). ADCs mostly decreased NCC MERP category B and D errors by 75%. The NCC MERP category C errors decreased by 43%. No errors resulted in patient deaths.

**Table 4:** Severity of medication errors based on the NCC MERP method for ADC items

NCC MERP Category	Pre	Post	P-value
B	4	1	1.000
C	7	4	0.635
D	4	1	1.000
Total	15	6	

ADC, automatic dispensing cabinet, NCC MERP, National Coordinating Council for Medication Error Reporting and Prevention

Pre: pre-ADC period, Post: post-ADC period

## Discussion

Based on our study results, the adoption of ADCs effectively reduced medication errors in the ICU: the dispensing error rate reduced from 3.87 to 0 per 100,000 dispensations (p = 0.003), and the prescription error rate reduced from 3.03 to 1.75 per 100,000 prescriptions (p = 0.500) for all items. The administrative error rate decreased from 0.046 to 0.026% for the ADC items (p = 0.424). Our study also found that ADCs were effective in reducing errors that caused no harm (NCC MERP categories B–D).

Dispensing is a high-risk activity because of the heavy workload of pharmacists [14]. A dispensing error can be defined as the discrepancy between a prescription and a medication dispensation to a patient. A systematic review showed that most reported dispensing errors were near misses, meaning that they were detected before leaving the pharmacy; the dispensing error rates ranged from 0.015 to 33.5% [15]. Preventing dispensing errors reduces the risk of drug-related harm or death to patients. However, there are several preventive measures for traditional manual dispensing, but they are unlikely to fully eliminate mechanical errors because of the potential for human error [2]. The adoption of ADCs and other automated medication distribution systems is one of the major solutions for promoting medication safety, and they have attracted attention in several developed countries [16]. ADCs allow the physical separation of similar medications, restrict medications access, and assist nurses in picking up medications through a guidance system [7]. A Swiss study comparing ADCs with traditional ward stocks found that ADCs can significantly reduce dispensing errors from 5 to 1% [17]. Another study conducted in Taiwan found that dispensing errors were reduced by 75% after the adoption of ADCs, even though ADCs were used to dispense prescriptions with only stat orders [5]. In the current study, ADCs were employed to dispense the most of commonly prescribed medications, accounting for approximately 80% of all dispensing volume. The remaining 20% were manually dispensed by pharmacists, and the greatly reduced workload might further contribute to the significantly decreased dispensing errors rate [18]. When taking into the 253 medications into account, the error events decreased from 2.45 to zero per 100,000 dispensations (p = 0.057), which might be deemed to have a trend toward statistical significance. The high accuracy of automated dispensing, combined with the reduced manual dispensing burden, tended to reduce the risk of human error for all items.

The primary function of the ADC is to ensure that medications are dispensed and delivered correctly. Therefore, ADCs can prevent the administration of an incorrect dose or strength to the patient so far as they are supplied correctly [19]. However, once the medication was removed, administration accuracy depended on the nurse. The adoption of ADCs did not consistently reduce the medication errors of nurses. Fanning et al. reported that the use of ADCs in emergency rooms reduced medication selection and preparation errors [7]. Chapuis et al. also reported that setting up ADCs in the ICU could effectively reduce medication errors related to preparation and administration [6]. In contrast, some studies have shown that ADCs have no significant effects on administrative errors [20, 21]. In our study, the use of ADCs reduced the rate of administration of wrong doses but not the administrative errors. More than a quarter of the errors were due to the wrong time of administration (6 of 22, 27.3%). One possible reason for these errors is that the pick-up time for ADC items was too long. The remaining administrative errors after ADC adoption were errors that could not be prevented, such as administration to the wrong patients, administration of wrong medications, or administration via the wrong routes. The number of unauthorized drug-related errors increased even after the introduction of ADCs. These errors were mainly related to inaccurate execution of the medication process or unfamiliarity with medications. Studies have demonstrated that sufficient medication knowledge can effectively prevent medication errors [7]. Therefore, improving relevant education and training for nursing may improve patient safety. Pharmacists should also participate in drug knowledge-related training to further reduce medication errors.

Medications prescription is the first step during the medication use process; however, only a few studies have investigated the impact of ADCs on prescription errors [22]. Because of the complexity of intensive care unit patients, studies have reported that pharmacist interventions have an important clinical impact in reducing prescription errors [23]. In our study, prescription errors were reduced from 3.03 to 1.75 per 100,000 prescriptions after the adoption of ADCs in intensive care units. Pharmacists had more time to spend on clinical services and more comprehensive reviews of prescriptions, which would allow the detection of more errors before prescriptions were processed [22, 24]. As patients admitted to the ICU are more vulnerable to medication errors and relevant adverse drug events, this could indirectly improve the quality of patient care by allowing pharmacists to spend more time on clinical work and improve medication safety [25].

Several errors were detected via other preventive measures before the patients received their medications; therefore, severe harm to patients during hospitalization was very rare [7]. In our study, all errors observed were classified as errors that did not cause harm (categories B–D). Chapuis et al. found that ADCs only reduced non-harmful errors and had no significant effect on harmful errors [6]. In this study, errors were caught through direct observation, with observers intervening when they were likely to cause patient harm. Therefore, potentially harmful errors were included in the study, even if they did not affect the patient. Most errors that could harm patients were related to nursing practices, such as administering medicataions at the wrong infusion rate or route. These mistakes cannot be prevented with the use of ADCs.

ADCs have reduced medication errors; however, some issues, ADC overrides due to verbal orders, need to be addressed. Cho et al. found that two-thirds of administrative errors were related to verbal orders [26]. They pointed out that verbal orders were among the main reasons for medication errors even after the implementation of the electronic hospital system. Since verbal orders are routine practice for Code Blue, pharmacists in the United States are responsible for preparing the medicine in the code car for patients to use [11]. The participation of clinical pharmacists would be valuable to improving medication safety during emergencies; however, this is currently not feasible in several countries. ADC overrides should be assessed periodically to prevent misuse. For example, the reason for each ADC override should be documented and the ADC override medication list should be periodically re-examined [27]. These measures require pharmacists and clinical medical staff discussions to determine the specific methods suitable for each hospital.

The major strength of our study was that it demonstrated the impact of ADC adoption on prescription errors, which has been relatively under-explored in previous studies. These types of errors were not directly related to ADCs, and the reallocation of pharmacist tasks and full review of prescriptions as a result of ADC adoption may have resulted in several indirect benefits. Our study has some limitations. First, the rate of medication errors was relatively low, and some trends may not have been discernible due to the small sample size of error events. The primary reason for the low reporting rate was that we did not report near-miss events related to prescription and dispensing errors. However, we reported every error that occurred and established incentives and penalties to prevent non-reporting of events. Second, because the observation period coincided with the COVID-19 outbreak, the baseline characteristics of the medical center may have been affected, which, in turn, may have affected the comparability of the data. However, based on our observations, we found that the characteristics of the ICU wards were similar during the two observation periods (Table 1). This may be attributed to the less severe epidemic in Taiwan than in other countries during that period; the epidemic did not significantly affect medical care in Taiwan, as expected. Third, our findings may not be generalizable to general wards. In addition, because the observation period spun only six months before and after ADC adoption, the medical and nursing staff may not have been completely familiar with the new operational processes. If the observation period was extended, the results are likely to be different. Therefore, further studies are needed to assess the impact of ADCs in other wards over longer periods. Additionally, using the Swiss Chess model or the work system for patient design (SEIPS) methology could identify the errors attributed to human mistakes.

## Conclusions

The adoption of ADCs has made closed-loop electronic medication management systems more integrated from medication prescription to administration. While medication errors were significantly reduced, possible human errors could not be eliminated with the ADC system. To further improve medication safety, multidisciplinary collaboration, education, and training programs, from a systemic perspective, are warranted.

## Electronic Supplementary Material

Below is the link to the electronic supplementary material.

Supplementary Material 1

Supplementary Material 2

Supplementary Material 3

Supplementary Material 4

## Data Availability

All data generated or analyzed during this study are included in this published article and its supplementary information files. Further inquiries can be directed to the corresponding author.

## Abbreviations

ADCs: automated dispensing cabinets
NCC MERP: National Coordinating Council for Medication Error Reporting and Prevention
NCKUH: National Cheng Kung University Hospital
ICUs: Intensive care units

## References

[1] Definition of medication errors. https://www.nccmerp.org/about-medication-errors.

[2] Tariq RA, Vashisht R, Sinha A, Scherbak Y. Medication dispensing errors and prevention. StatPearls. Treasure Island (FL): StatPearls Publishing. Copyright. StatPearls Publishing LLC. 2022. 30085607

[3] Prgomet M, Li L, Niazkhani Z, Georgiou A, Westbrook JI (2017). Impact of commercial computerized provider order entry (CPOE) and clinical decision support systems (CDSSs) on medication errors, length of stay, and mortality in intensive care units: a systematic review and meta-analysis. J Am Med Inform Assoc.

[4] Austin JA, Smith IR, Tariq A (2018). The impact of closed-loop electronic medication management on time to first dose: a comparative study between paper and digital hospital environments. Int J Pharm Pract.

[5] Wang E-Y, Hu H-Y, Hong Y-C, Ai M-Y, Chen T-J, Chen Y-C (2021). Implementation of automated dispensing cabinet to improve process of inpatient drug delivery. Formosa. J Clin Pharm.

[6] Chapuis C, Roustit M, Bal G, Schwebel C, Pansu P, David-Tchouda S (2010). Automated drug dispensing system reduces medication errors in an intensive care setting. Crit Care Med.

[7] Fanning L, Jones N, Manias E (2016). Impact of automated dispensing cabinets on medication selection and preparation error rates in an emergency department: a prospective and direct observational before-and-after study. J Eval Clin Pract.

[8] Risør BW, Lisby M, Sørensen J (2018). Complex automated medication systems reduce medication administration errors in a Danish acute medical unit. Int J Qual Health Care.

[9] Franklin BD, O’Grady K, Donyai P, Jacklin A, Barber N (2007). The impact of a closed-loop electronic prescribing and administration system on prescribing errors, administration errors and staff time: a before-and-after study. Qual Saf Health Care.

[10] Koehler T, Brown A (2017). A global picture of pharmacy technician and other pharmacy support workforce cadres. Res Social Adm Pharm.

[11] Merchan C, Soliman J, Ahuja T, Arnouk S, Keeley K, Tracy J (2020). COVID-19 pandemic preparedness: A practical guide from an operational pharmacy perspective. Am J Health Syst Pharm.

[12] Barker KN, Flynn EA, Pepper GA, Bates DW, Mikeal RL (2002). Medication errors observed in 36 health care facilities. Arch Intern Med.

[13] Taxonomy of medication errors. https://www.nccmerp.org/taxonomy-medication-errors-now-available.

[14] James KL, Barlow D, McArtney R, Hiom S, Roberts D, Whittlesea C (2009). Incidence, type and causes of dispensing errors: a review of the literature. Int J Pharm Pract.

[15] Aldhwaihi K, Schifano F, Pezzolesi C, Umaru N (2016). A systematic review of the nature of dispensing errors in hospital pharmacies. Integr Pharm Res Pract.

[16] Ahtiainen HK, Kallio MM, Airaksinen M, Holmström AR (2020). Safety, time and cost evaluation of automated and semi-automated drug distribution systems in hospitals: a systematic review. Eur J Hosp Pharm.

[17] Jumeau M, Francois O, Bonnabry P. Impact of automated dispensing cabinets on dispensing errors, interruptions and pillbox preparation time. Eur J Hosp Pharm. 2021. 10.1136/ejhpharm-2021-002849 PMC10359777 34426488

[18] Irwin A, Ross J, Seaton J, Mearns K (2011). Retrospective analysis of DATIX dispensing error reports from Scottish NHS hospitals. Int J Pharm Pract.

[19] Institute of Medicine Committee on Quality of health care in A. In. In: Kohn LT, Corrigan JM, Donaldson MS, editors. To err is human: building a safer health system. Washington (DC): National Academies Press (US) Copyright; 2000. p. 2000 by the National Academy of Sciences. All rights reserved. 25077248

[20] Dib JG, Abdulmohsin SA, Farooki MU, Mohammed K, Iqbal M, Khan JA (2006). Effects of an automated drug dispensing system on medication adverse event occurrences and cost containment at SAMSO. Hosp Pharm.

[21] Cottney A. Improving the safety and efficiency of nurse medication rounds through the introduction of an automated dispensing cabinet. BMJ Qual Improv Rep. 2014;3. 10.1136/bmjquality.u204237.w1843 PMC4645698 26734256

[22] Barber N, Cornford T, Klecun E (2007). Qualitative evaluation of an electronic prescribing and administration system. Qual Saf Health Care.

[23] Bourne RS, Shulman R, Jennings JK (2018). Reducing medication errors in critical care patients: pharmacist key resources and relationship with medicines optimisation. Int J Pharm Pract.

[24] Tsao NW, Lo C, Babich M, Shah K, Bansback NJ (2014). Decentralized automated dispensing devices: systematic review of clinical and economic impacts in hospitals. Can J Hosp Pharm.

[25] Klopotowska JE, Kuiper R, Van Kan HJ, De Pont AC, Dijkgraaf MG, Lie-A-Huen L (2010). On-ward participation of a hospital pharmacist in a Dutch intensive care unit reduces prescribing errors and related patient harm: an intervention study. Crit Care.

[26] Cho I, Park H, Choi YJ, Hwang MH, Bates DW (2014). Understanding the nature of medication errors in an ICU with a computerized physician order entry system. PLOS ONE.

[27] Cello R, Conley M, Cooley T, De la Torre C, Dorn M, Ferer DS (2022). ASHP guidelines on the safe use of automated dispensing cabinets. Am J Health Syst Pharm.

