# Supplementary material for: Reducing Medication Errors by Adopting Automatic Dispensing Cabinets in Critical Care Units
Source: J Med Syst. 2023 Apr 27;47(1):52. doi: 10.1007/s10916-023-01953-0 (PMC10136387; doi:10.1007/s10916-023-01953-0)
Supplement: Supplementary file 1 — Supplementary Material 1 [file 10916_2023_1953_MOESM1_ESM.docx]

| Additional File 1. ATC code of items in ADC | | |
| --- | --- | --- |
| ATC code category | N | % |
| Cardiovascular system | 52 | 20.55 |
| Alimentary tract and metabolism | 43 | 17.00 |
| Anti-infectives for systemic use | 36 | 14.23 |
| Blood and blood-forming organs | 34 | 13.44 |
| Nervous system | 31 | 12.25 |
| Respiratory system | 15 | 5.93 |
| Systemic hormonal preparations, excl. Sex hormones and insulins | 13 | 5.14 |
| Musculoskeletal system | 7 | 2.77 |
| Dermatological | 7 | 2.77 |
| Various | 4 | 1.58 |
| Sensory organs | 4 | 1.58 |
| Antineoplastic and immunomodulating agents | 3 | 1.19 |
| Genitourinary system and sex hormones | 3 | 1.19 |
| Antiparasitic products, insecticides, and repellents | 1 | 0.40 |
| Total | 253 | 100.00 |

ATC: anatomical therapeutic chemical classification; ADC: automatic dispensing cabinet

Note: Those medications selected for ADC dispensing were based on the consumption volume and prescription frequency. On the other hand, medications that were classified as the controlled substances or required refrigerator storage were excluded due to the hardware limitation.
