# Supplementary material for: Reducing Medication Errors by Adopting Automatic Dispensing Cabinets in Critical Care Units
Source: J Med Syst. 2023 Apr 27;47(1):52. doi: 10.1007/s10916-023-01953-0 (PMC10136387; doi:10.1007/s10916-023-01953-0)
Supplement: Supplementary file 2 — Supplementary Material 2 [file 10916_2023_1953_MOESM2_ESM.docx]

| Additional File 2. Definitions of medication errors per Barker et al. (12) | |
| --- | --- |
| Category | Description of category |
| Unauthorized drug | The administration of a dose of medication that had never been ordered for that patient (wrong patient, wrong drug). |
| Extra dose | Any dose administered in excess of the total number of times ordered by the physician. |
| Wrong dose | Any dose of performed dosage units that contained the wrong strength or number. |
| Omission | Failure to give an ordered dose. |
| Wrong route | Medication administered to a patient using a different route than ordered. |
| Wrong form | The administration of a dose in a different form than ordered. |
| Wrong technique | Exclusion, or incorrect performance, of a procedure ordered by the prescriber immediately before administration of each dose of medication. |
| Wrong time | Administration of a dose more than 60 minutes before or after the scheduled administration time. |
