# Supplementary material for: Reducing Medication Errors by Adopting Automatic Dispensing Cabinets in Critical Care Units
Source: J Med Syst. 2023 Apr 27;47(1):52. doi: 10.1007/s10916-023-01953-0 (PMC10136387; doi:10.1007/s10916-023-01953-0)
Supplement: Supplementary file 3 — Supplementary Material 3 [file 10916_2023_1953_MOESM3_ESM.docx]

| Additional File 3. NCC MERP Definition of medication error severity (13) | |
| --- | --- |
| Category | Description of category |
| No error |  |
| A | Circumstances or events that can cause errors. |
| Error, no harm |  |
| B | An error occurred but the medication did not reach the patient. |
| C | An error occurred that reached the patient but did not cause patient harm. |
| D | An error occurred that resulted in the need for increased patient monitoring but no patient harm. |
| Error, harm |  |
| E | An error occurred that resulted in the need for treatment or intervention and caused temporary patient harm. |
| F | An error occurred that resulted in initial or prolonged hospitalization and caused temporary patient harm. |
| G | An error occurred that resulted in permanent patient harm. |
| H | An error occurred that resulted in a near-death event (e.g., anaphylaxis, cardiac arrest. |
| Error, death | An error occurred that resulted in patient death. |

NCC MERP, National Coordinating Council for Medication Error Reporting and Prevention
