# Supplementary material for: Reducing Medication Errors by Adopting Automatic Dispensing Cabinets in Critical Care Units
Source: J Med Syst. 2023 Apr 27;47(1):52. doi: 10.1007/s10916-023-01953-0 (PMC10136387; doi:10.1007/s10916-023-01953-0)
Supplement: Supplementary file 4 — Supplementary Material 4 [file 10916_2023_1953_MOESM4_ESM.docx]

| Additional File 4. List of medication errors for ADC items | | | | |
| --- | --- | --- | --- | --- |
| Error event | Period | Stage | Type of error | Prevented by ADC |
| Intravenous nicardipine, wrong dose (verbal order) | Post-ADC | Prescribing | Wrong dose | No |
| Intravenous dopamine administrated to the wrong patient | Post-ADC | Administration | Unauthorized drug | No |
| Intravenous metoclopramide, omission of medication | Post-ADC | Administration | Omission | No |
| Intravenous labetalol instead of nicardipine | Post-ADC | Administration | Unauthorized drug | No |
| Inhaled tranexamic acid administrated by intravenous route | Post-ADC | Administration | Route | No |
| Intravenous ampicillin/sulbactam, omission of medication | Post-ADC | Administration | Omission | No |

ADC, automatic dispensing cabinet, Post-ADC, post-ADC period
